# Hepatic angiosarcoma arising in an adult mesenchymal hamartoma

**DOI:** 10.1186/1477-7800-4-3

**Published:** 2007-01-26

**Authors:** Qiang Li, Jian Wang, Yan Sun, Yunlong Cui, Xishan Hao

**Affiliations:** 1 Department of Hepatobiliary Surgery, Cancer Hospital of Tianjin Medical University, Huanhu Western Road, Hexi District, Tianjin 300060, China PR; 2 Department of Pathology, Cancer Hospital of Tianjin Medical University, Huanhu Western Road, Hexi District, Tianjin 300060, China PR

## Abstract

The histogenesis of the hepatic sarcoma and its association with hamartoma is not well understood. We hereby present a Chinese patient with hepatic angiosarcoma arising from an adult mesenchymal hamartoma of liver. A 33-yr-old woman was diagnosed hepatic hamartoma eight years ago and presented with epigastric distention recently. Now she was admitted to our hospital with some unusual features: (a) this patient was diagnosed in mid-twenties, (b) the tumor occupied the whole liver and most importantly (c) the hepatic angiosarcoma appeared 8 years after the diagnosis of hamartoma. Based on this case and some reports, hepatic hamartoma may develop to hepatic angiosarcoma.

## Background

Mesenchymal hamartoma, mostly seen in young infants and exceptionally in adult patients, is a rare and benign developmental tumour of the liver. It usually presents as a large and multicystic mass of loose and sometimes nodular mesenchymal tissue. This tumor is thought to result from a developmental anomaly possibly related to abnormal vascular supply [1]. Hepatic angiosarcoma, however, is the most common primary sarcoma of the liver, usually absence of curable treatments and poor prognosis [2]. The histogenesis of the hepatic undifferentiated sarcoma and its association with hamartoma has been much debated. We herein report one case of hepatic angiosarcoma arising from an adult hepatic mesenchymal hamartoma.

## Case report

### Clinical findings

A 33-yr-old woman was admitted to Department of Hepatobiliary Surgery at Cancer Hospital of Tianjin Medical University, China, with the diagnosis of hepatic hamartoma. Eight years previously, she was diagnosed with hepatic hamartoma by fine needle biopsy. She did not accept effective treatment, and subsequently presented with epigastric distention. A history of hepatitis (HBV, HCV) and hepatic cirrhosis were excluded with appropriate tests. Physical examination revealed slight paleness of the skin and mucosa and no jaundice. Abdominal palpation demonstrated the presence of shifting dullness and hepatomegaly without obvious tenderness. No other abnormal signs were found.

### Laboratory tests and Imaging

The patients' blood results were as follows:

Hematology; RBC: 3.64 × 10^12^/L, Hb: 61 g/L, WBC: 4.40 × 10^9^/L and PLT: 126 × 10^9^/L. Biochemistry; showed: ALT 23 U/L, AST 35 U/L, ALP 386 U/L, ALB 28.0 g/L, TP 50.4 g/L, GLB 22.4 g/L and GGT 254 U/L. Serum markers for HBV and HCV were negative. AFP, CA125, CA199 and CEA were normal.

**Figure 1:**
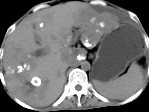
Unenhanced spiral CT scan showed liver with rugositied surface, multinodular focuses involved the whole liver, calcified plaques and ascites.

Both ultrasonography and spiral computerized tomography (CT) revealed an enlarged liver with rugositied surface. Multinodular focuses involved the whole liver accompanied with calcified plaques, cholecystolithiasis, multiple tiny nodules under the peritoneum and ascites (figure 1).

### Management and pathology

After a multidisciplinary consensus, the patient underwent surgery including omentectomy and excisional biopsy of liver tumors. In the operation, asymmetrical nodules were noticed on the whole liver and beneath the peritoneum. One was located at colic omentum with 1.5 cm diameter. Frozen section biopsy revealed angiosarcoma. Therefore, only colic omentum resection and hepatic multi-point biopsy were performed.

**Figure 2:**
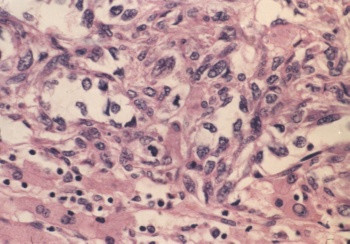
Haematoxylin and eosin stain (×400); typical features of hepatic angiosarcoma including sinusoidal and spindle-shape growth of the malignant endothelial cells, atrophy of liver cells and disruption of the hepatic plates.

**Figure 3:**
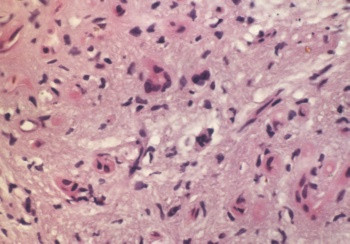
Haematoxylin and eosin stain (×400); mesenchymal hamartoma with spindle and stellate cells in the mucoid matrix.

**Figure 4:**
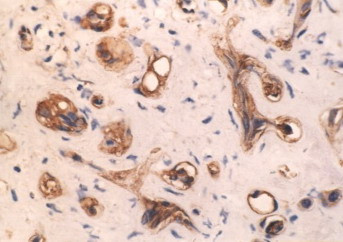
Immunohistochemistry of CD34 in angiosarcoma (streptavidin peroxidase method, ×400) immunoreactivity mainly localized to the cytoplasm of malignant cells.

**Figure 5:**
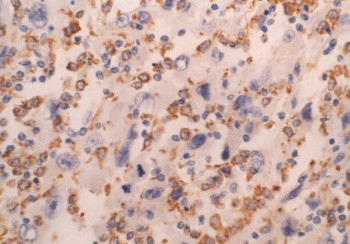
Immunohistochemistry of Vimentin in angiosarcoma (streptavidin peroxidase method, ×400) immunoreactivity mainly localized to the cytoplasm of malignant cells.

Postoperative pathology revealed angiosarcoma (figure 2) arising from hepatic hamartoma (figure 3) accompaniedwith coelio-implantation metastasis. Subsequent immunohistochemistry revealed the lesions were CD34(+) (figure 4), Vimentin(+) (figure 5), S-100(-), CEA(-) and CK(-) (Zymed).

The patient survived 4 months after surgery, and died from liver failure.

## Discussion

Mesenchymal hamartoma appears as a disordered arrangement of mesenchyme, bile ducts, and hepatic parenchyma in histology. Grossly, it has stromal and cystic components with no capsule and can grow to large sizes (16 cm being an average tumor size).

Clinically, patients with mesenchymal hamartoma tend to present in the first 2 years of life, with a median age of 10 months (0–19 years). The right lobe is more frequently affected than the left (6: 1) [1]. Typical presentation is one of asymptomatic, rapid abdominal distension with a palpable mass on physical examination. The radiological appearance is one of a large, uni- or multicystic, avascular mass occupying part of the liver.

Surgical resection has been the standard treatment for this tumor. Although our patient had some typical clinical and radiological picture of mesenchymal hamartoma, there were three unusual features: she was diagnosed at a later age (25 years old), the tumor occupied the whole liver, and hepatic angiosarcoma arised 8 years after the diagnosis of hamartoma.

Hepatic angiosarcoma is frequently associated with environmental carcinogens such as thorotrast, vinyl chloride and arsenic compounds [3,4]. There was no evidence of carcinogen exposure and extrahepatic angiosarcoma in our patient, which led us to question whether this tumor was an evolution from pre-existing mesenchymal hamartoma in this patient.

The association between undifferentiated sarcoma and hamartoma of the liver has been much debated. Recently, discovery of a similar genetic abnormality in both lesions has supported the supposed link between them. Lauwers et al. reported a case of a hepatic undifferentiated (embryonal) sarcoma arising within a mesenchymal hamartoma in a 15-year-old girl with the histologic, flow cytometric, and cytogenetic evidence [5]. de Chadarevian [6]and O'Sullivan [7] also reported cases of undifferentiated embryonal sarcoma arising from mesenchymal hamartoma with the features of histology and immunohistochemistry (cytokeratins, alpha-1-antitrypsin and vimentin), respectively.

Clinciains should be aware that hepatic angiosarcoma may develop from pre-existing hepatic hamartoma. Timely management of these lesions is important to prevent any possible malignant evolution.
